# Chocolate, gut microbiota, and human health

**DOI:** 10.3389/fphar.2013.00011

**Published:** 2013-02-07

**Authors:** Nabil Hayek

**Affiliations:** Department of Biochemistry, Microbiology and Immunology, University of OttawaOttawa, ON, Canada

**Figure 1:**
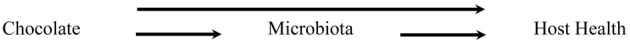
**The effect of chocolate on human health could be mediated by its effect on the gut microbiota that ultimately lead to changes in host's health**.

With the advances in molecular biology techniques, the association between changes in the gut microbiota and human diseases or disorders is becoming more evident. These health issues include aging (Rehman, 2012), oxidative stress (Qiao et al., 2012), blood pressure and atherosclerosis (Queipo-Ortuño, 2012), diabetes (Wen et al., 2008; Larsen et al., 2010); cancer (Mai et al., 2007), and different other central nervous system disorders (Diaz Heijtz, 2011). By the same token, cocoa was shown to affect the same human disorders that were linked to gut microbiota. Together, these findings could imply that chocolate or cocoa could exert its effect by altering the gut microbiota (Figure 1). Based on this observation, the effect of chocolate on the gut microbiota will be discussed further in this short article.

Ingredients in diets that are derived from prebiotics and consumed as probiotics or synbiotics play a role in changing the human gut microbiota (Steer et al., 2000). Chocolate or cocoa is considered a prebiotic that is rich, among other chemicals, in polyphenols (Redovniković et al., 2009). These polyphenols are flavonoids in which procyanidins, like catechin and epicatechin oligomers, constitute the majority of the proanthocyanidin member in this class. Therefore, terms such as polyphenols, cocoa, and gut microbiota were used to retrieve relevant articles in PubMed and Google Scholar. Even though the number of articles was less than 10 in PubMed, the effect of flavanols in cocoa on gut microbiota was assessed in recent studies.

For instance, in a controlled, double-blind, randomized clinical trial, researchers compared the outcomes of consuming a high-cocoa vs. a low-cocoa flavanol drinks (Tzounis et al., 2011). Their results show a significant increase in certain gut microbes such as Bifidobacteria and Lactobacilli. On the other hand, a significant decrease in Clostridia, which are a class of the Firmicutes, was noted. According to the same study, these changes in bacterial population was accompanied by changes in other biological markers such as triglyceride and C-reactive protein concentration.

A more recent study evaluated the minimum inhibitory concentration (MIC) of several polyphenols on selective human intestinal microbiota species (Duda-Chodak, 2012). The obtained data indicate that polyphenols in their aglycone form inhibit the growth of the tested microbial species. Whereas, their related glycosides did not affect bacterial viability. In addition, the catechin, which is one of the main polyphenols in chocolate and which is available only as an aglycone, did not have an inhibitory effect in contrast to other selected polyphenols. This result supports the findings of the previous study in which an increase in the Bifidobacteria and Lactobacilli was observed due to consuming a drink that is rich in cocoa. The other important observation is the fact that the interaction between the polyphenols and the gut microbiota is bidirectional. This means that gut microbes affect the hydrolysis and hence the absorption of the polyphenols and at the same time the products of this hydrolysis affect the growth of bacterial species that are present in the intestine either negatively or positively. In animals, the effect of cocoa was investigated on rat gut microbiota and the results were similar to that on human gut microbiota (Massot-Cladera et al., 2012). Massot-Caldera et al. reported a relationship between ingestion of cocoa, changes of the rat intestinal microbiota, and the intestinal immune response. This alteration in the intestinal ecosystem affected the expression of the intestinal Toll-like receptors (TLRs) and led to a lower level of Immunoglobulin A (IgA) secretion in the rats' intestines. As in the study that was conducted by Tzounis et al., this group found a significant decrease in Bacteroides, Clostridium, and Staphylococcus species in the faeces of rats that were on a cocoa diet.

Similarly, the effect of prebiotics on gut microbiota was extensively discussed in a recent article by Roberfroid et al. (2010). In their review the authors highlighted studies demonstrating, for example, differences between the microbiota ecosystem of patients with Irritable Bowel Syndrome (IBS) and healthy individuals. In all these studies, IBS subjects had lower numbers of Bifidobacteria, Lactobacilli, and a higher number of Clostridia. The potential effect of chocolate, therefore, as shown in Tzounis et al. could be evident in this case since it led to the increase in the Bifidobacteria and Lactobacilli population and a reduction in Clostridia.

Moreover, the authors classified the main bacterial phyla and genera in the gut on a scale indicating their abundance in the faeces and their potential harmful or beneficial effect. According to this scheme, Bifidobacteria and Lactobacilli were among the beneficial bacteria, whereas Clostridia was part of the harmful phyla.

To conclude, the effect of cocoa on the intestinal microbial ecosystem mimics the effect of prebiotics and probiotics on this system. As mentioned earlier, changes in the microbiota could affect the health status of the host. Chocolate was shown to play a role in different human diseases and disorders and its role could be through modulations of the intestinal microbial species as demonstrated in recent published studies.
